# Exploring Early Autism Markers in High-Risk Infants: Implications for Timely Intervention

**DOI:** 10.1192/j.eurpsy.2024.941

**Published:** 2024-08-27

**Authors:** M. Negm, N. Khoweiled

**Affiliations:** ^1^Health Education England, Birmingham and Solihull Mental Health Foundation Trust, Birmingham, United Kingdom; ^2^University of Strasbourg, Strasbourg, France

## Abstract

**Introduction:**

Autism Spectrum Disorder (ASD) is a neurodevelopmental condition characterized by challenges in social communication and behaviour. Timely identification of ASD is pivotal for effective intervention. However, significant gaps persist in our understanding of early signs and biomarkers, particularly among infants with older siblings already diagnosed with ASD. Furthermore, factors during the perinatal and neonatal period remain underexplored.

**Objectives:**

This systematic review aims to investigate early autism markers within this specific cohort and assess their potential impact on intervention strategies.

**Methods:**

A thorough search of electronic databases, including PubMed, PsycINFO, and Scopus, was conducted, initially identifying 161 relevant papers related to ASD and resilience published from 2013 to 2023. After excluding studies focused on environmental determinants of resilience in ASD, 75 papers remained. We concentrated on studies examining early identification of autism, especially in infants with older siblings with ASD, biomarker discovery, or predictive factors within this unique population. The search strategy employed a diverse set of keywords encompassing ASD, genetics, neurobiology, and the perinatal period to ensure comprehensive coverage of pertinent studies. Quality assessment of each study followed standardized criteria, and data synthesis utilized a thematic analysis approach.

**Results:**

Our systematic exploration revealed a spectrum of early markers associated with ASD in high-risk infants, spanning behavioural, neurodevelopmental, genetic, and perinatal domains. Recognizing these early indicators offers promise for timely and potent intervention strategies, potentially refining long-term outcomes for children at risk of ASD.

**Discussion:**

The synthesis of existing research in this systematic review underscores the significance of studying early markers within high-risk populations. Early intervention, guided by these markers, holds the potential to enhance the quality of life for at-risk children with ASD and their families. This review contributes to our understanding of the early identification of autism and emphasizes the imperative need for continued research in this critical area.

**Conclusions:**

This systematic review sheds light on the current state of research on early signs and biomarkers of autism in infants with older siblings diagnosed with ASD. The findings carry significant implications for the development of targeted interventions that can be implemented at an earlier stage of development. Future research should further investigate these markers and their potential role in guiding early and effective intervention strategies.

Keywords: Autism Spectrum Disorder, early signs, biomarkers, infants, older siblings, early intervention, high-risk population.

**Disclosure of Interest:**

None Declared

